# Phytochemicals in Cancer Treatment: From Preclinical Studies to Clinical Practice

**DOI:** 10.3389/fphar.2019.01614

**Published:** 2020-01-28

**Authors:** Amit S. Choudhari, Pallavi C. Mandave, Manasi Deshpande, Prabhakar Ranjekar, Om Prakash

**Affiliations:** ^1^ Combi-Chem Bio-Resource Center, Organic Chemistry Division, CSIR-National Chemical Laboratory, Pune, India; ^2^ Interactive Research School of Health Affairs, Bharati Vidyapeeth Deemed University, Pune, India; ^3^ Department of Dravyaguna Vigan, Ayurved Pharmacology, College of Ayurved, Bharati Vidyapeeth Deemed University, Pune, India; ^4^ Innovation Biologicals Pvt. Ltd., Pune, India; ^5^ Department of Microbiology, Immunology and Parasitology, Louisiana State University Health Sciences Center, New Orleans, LA, United States; ^6^ Stanley S. Scott Cancer Center, Louisiana State University Health Sciences Center, New Orleans, LA, United States

**Keywords:** phytochemicals, anticancer, preclinical, clinical, medicinal plants

## Abstract

Cancer is a severe health problem that continues to be a leading cause of death worldwide. Increasing knowledge of the molecular mechanisms underlying cancer progression has led to the development of a vast number of anticancer drugs. However, the use of chemically synthesized drugs has not significantly improved the overall survival rate over the past few decades. As a result, new strategies and novel chemoprevention agents are needed to complement current cancer therapies to improve efficiency. Naturally occurring compounds from plants known as phytochemicals, serve as vital resources for novel drugs and are also sources for cancer therapy. Some typical examples include taxol analogs, vinca alkaloids such as vincristine, vinblastine, and podophyllotoxin analogs. These phytochemicals often act *via* regulating molecular pathways which are implicated in growth and progression of cancer. The specific mechanisms include increasing antioxidant status, carcinogen inactivation, inhibiting proliferation, induction of cell cycle arrest and apoptosis; and regulation of the immune system. The primary objective of this review is to describe what we know to date of the active compounds in the natural products, along with their pharmacologic action and molecular or specific targets. Recent trends and gaps in phytochemical based anticancer drug discovery are also explored. The authors wish to expand the phytochemical research area not only for their scientific soundness but also for their potential druggability. Hence, the emphasis is given to information about anticancer phytochemicals which are evaluated at preclinical and clinical level.

## Introduction

Cancer is a major public health problem that has a significant global impact on both developed and developing countries. In 2018, an estimated 18.1 million new cases of cancer occurred worldwide which are likely to increase to 23.6 million new cases each year by 2030 (Bray et al., 2018). Considering the high profile nature of the disease, its treatment has been a constant struggle with relatively less success. Currently available options for cancer treatment involve surgical removal and radiation treatment of the large accumulated biomass of cancer, typically followed by systemic chemotherapy treatment for maintenance. The primarily available chemotherapeutic agents include antimetabolites (e.g., methotrexate), DNA-interactive agents (e.g., cisplatin, doxorubicin), anti-tubulin agents (taxanes), hormones, and molecular targeting agents (Nussbaumer et al., 2011). The major disadvantages of chemotherapy are recurrence of cancer, drug resistance, and toxic effects on non-targeted tissues that can restrain the use of anticancer drugs and thus impair patient’s quality of life. To overcome the problems of present therapy, search for new promising anticancer agents with better efficacy and lesser side effects continues.

Phytochemicals and derivatives present in plants are promising options to improve treatment efficiency in cancer patients and decrease adverse reactions. A number of these phytochemicals are naturally occurring biologically active compounds with significant antitumor potential. The development of effective and side-effects free phytochemical based anticancer therapy begins with the testing of natural extracts (from dry/wet plant material) for potential anticancer biological activity followed by purification of active phytochemicals based on bioassay-guided fractionation and testing for *in vitro* and *in vivo* effects. In the present review, an attempt has been made to gather information specifically about the anti-cancer phytochemicals that are evaluated at preclinical and clinical levels as well as those available in the market, until now. In preclinical section, we have reviewed the phytochemicals with a reported *in vivo* activity. This review further highlights phytochemicals which are assessed at preclinical level and also mentions some phytochemicals which are in the clinical trials along with the brief information on the presently used plant-based anticancer drugs.

## Plant Derived Drugs: A Historical Perspective

Plants have been used to treat various disease aliment from time immemorial. Ayurveda, Traditional Indian Medicine (TIM), and the Traditional Chinese Medicine (TCM) remain the most ancient (4500 BC) yet living traditions. In the ancient period, the knowledge of selection of right plants, a specific time for their collection, method of drug preparation with their specific use was transferred verbally from one generation to the next generation. The folklore system has documented all parameters about the drugs and their specific uses in the disease conditions. These drugs were prepared as tinctures, teas, powders, poultices, decoctions, and other types of formulations (Ogbonna et al., 2012; Fridlender et al., 2015) which were the most common methods of drug preparation until 18^th^ century. Unfortunately, none of them could fit into the modern scientific definition of a drug.

With advances in organic chemistry and chemical analysis, an analytical investigation of active components of medicinal plants and herbal remedies was pursued in late 18^th^ or early 19^th^ centuries, which opened the doors toward the isolation/purification and characterization of numerous active principles of plants. This increased the pace of drug discovery and led to a miracle innovation in the medical field. The first breakthrough which launched the first generation of drugs came with the isolation of analgesic (pain killing) drugs morphine from the plant *Papaver somniferum*. Later, many well-defined 20^th^ century drugs were derived from plants, including salicylic acid, the precursor of aspirin (*Salix* sp.), cocaine (*Erythroxylum coca*), quinine (*Cinchona officinalis*), digitoxin (*Digitalis purpurea* and *Digitalis lanata)*, and many others with pharmaceutical and clinical potential (Newman et al., 2000; Butler, 2004; Ogbonna et al., 2012). Over the period from around 1981 to the end of 2014, more than half of all approved small‐molecule drugs originated from natural products, where they served as drug precursors, templates for synthetic modification, and pharmacological probes (Newman and Cragg, 2016). This in itself demonstrates the enormous medicinal potential of plants that has been known for thousands of years in traditional medicine. A few commercially available plant-derived compounds used against various diseases are listed in **Table 1**.

**Table 1:** List of commercially available plant derived drugs on various diseases.

Drug	Class of drug	Plant source	Disease	Reference
Apomorphine	Dopamine receptor agonist	*Papaver somniferum L.*	Parkinson	(Deleu et al., 2004)
Arteether	Sesquiterpene trioxane lactone	*Artemisia annua*	Malaria	(van Agtmael et al., 1999)
Galantamine	Amaryllidaceae alkaloid	*Galanthus woronowii*	Alzheimer	(Heinrich and Lee Teoh, 2004)
Nitisinone	Mesotrione	*Callistemon citrinus*	Hepatorenal tyrosinemia	(Das, 2017)
Paclitaxel	Taxane diterpene	*Taxus brevifolia* Nutt.	Cancer	(Wani et al., 1971)
Tiotropium	Muscarinic receptor antagonist	*Atropa belladonna*	Asthma and COPD	(Mundy and Kirkpatrick, 2004)

COPD, chronic obstructive pulmonary disease.

## Phytochemicals With Anticancer Properties

Scientific evidences indicate that phytochemicals have significant antitumor potential. Approximately, 50% of approved anticancer drugs from 1940 to 2014 originate from natural products or directly derived therefrom (Newman and Cragg, 2016). Some of the remarkable anticancer phytochemicals in this regard are describe in the present review. These phytochemicals have been tested for anti-cancer efficacy at both *in vitro* and *in vivo* levels. They possess complementary and overlapping mechanisms to slow down the carcinogenic process by scavenging free radicals (Lee et al., 2013), suppressing survival and proliferation of malignant cells (Yan et al., 2018), as well as diminishing invasiveness and angiogenesis of tumors (Lu et al., 2018a). They exert wide and complex range of actions on different molecular targets and signal transduction pathways including membrane receptors (Deng et al., 2017), kinases (Dou et al., 2018), downstream tumor-activator or -suppressor proteins (Adams et al., 2010), transcriptional factors (Zhang et al., 2017b), microRNAs (miRNAs) (Cojocneanu et al., 2015), cyclins, and caspases (Yan et al., 2018).

## Phytochemicals in Pre-Clinical Trials

**Figure 1:**
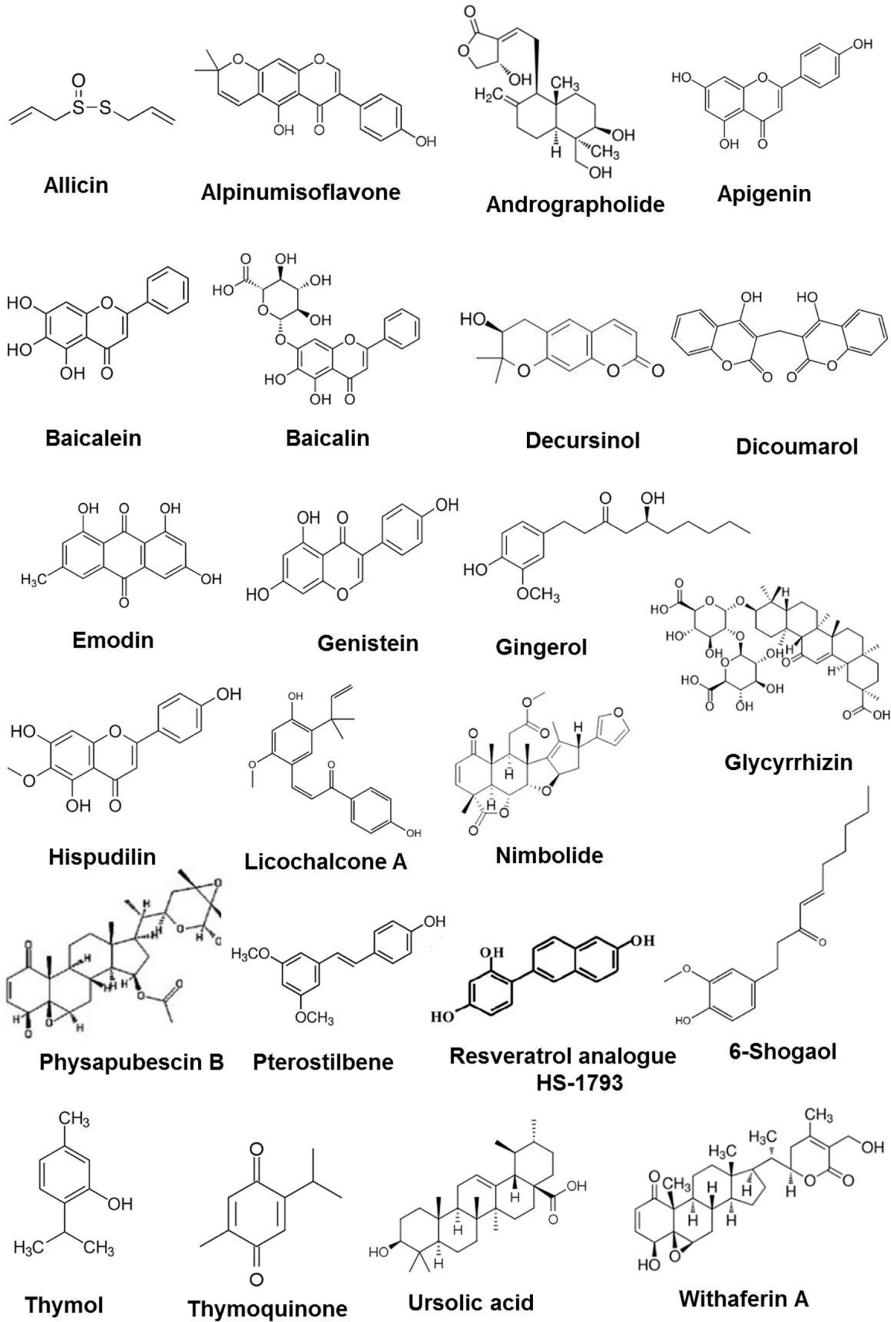
Chemical structures of some anticancer phytochemicals in preclinical trials.

In a bench to bedside drug development process, meticulous use of preclinical screening models can results into potential lead compounds for anticancer drug development with extensive data on preliminary efficacy, toxicity, pharmacokinetic, and safety information which help to decide whether a molecule should be taken further for clinical trials. In the context of this review, abundant evidence has been collected on preclinical efficacy of number of phytochemicals (**Figure 1**) in various animal models which is summarized in **Table 2**. Brief information on each phytochemical is as follows:

**Table 2:** Phytochemicals in pre-clinical trials for cancer treatment.

Phytochemical (compound class)	Botanical name (family)	Molecular targets	Reference
6-Shogaol (phenylpropanoid)	*Zingiber officinale* (Roscoe)	Akt and STAT signaling pathway	(Kim et al., 2014; Saha et al., 2014)
Allicin (organosulfurs)	*Allium sativum* (Amaryllidaceae)	STAT3 signaling pathway	(Huang et al., 2017; Chen et al., 2018)
Alpinumisoflavone (pyranoisoflavone)	*Derris eriocarpa* (Leguminosae)	Nrf2, NQO-1, HO-1, miR-101, and Akt signaling	(Wang et al., 2017; Zhang et al., 2017a)
Andrographolide (diterpenoid)	*Andrographis paniculata* (Acanthaceae)	HIF-1α, VEGF, and PI3K pathway	(Li et al., 2015)
Apigenin (flavonoid)	*Petroselinum crispum* (Apiaceae)	Intrinsic apoptosis pathway	(Chang et al., 2018; Yan et al., 2018)
Baicalein (flavonoid)	*Scutellaria baicalensis* (Lamiaceae)	MAPK, ERK, and p38 signaling pathways	(Dou et al., 2018; Tao et al., 2018)
Baicalin (flavonoid)	*Scutellaria baicalensis* (Lamiaceae)	MAPK, ERK, and p38 signaling pathways	(Dou et al., 2018)
Curcumin (phytopolyphenol)	*Curcuma longa* (Zingiberaceae)	Modulates cell signaling and gene expression regulatory pathways	(Kunnumakkara et al., 2017)
Decursin and Decursinol (Coumarin)	*Angelica gigas* (Apiaceae)	Not mentioned	(Wu et al., 2017)
Dicumarol	*Melilotus officinalis* (Fabaceae)	Intrinsic apoptosis pathway	(Zhang et al, 2017b)
Epigallocatechin (flavonoids)	*Camellia sinensis* (Theaceae)	Inhibit cell proliferation and apoptosis	(Xu et al., 1992; Thangapazham et al., 2007)
Emodin (resin)	*Rheum palmatum* L. (Polygonaceae)	PI3K/AKT and MAPK signaling pathways	(Iwanowycz et al., 2016; Lin et al., 2016; Su et al., 2017)
Genistein (isoflavonoid)	*Glycine max* (legumes)	WNT/β-catenin and Akt signaling pathway	(Zhang et al., 2013; Hsiao et al., 2019)
Gingerol (polyphenol)	*Zingiber officinale* (Roscoe)	Intrinsic apoptosis pathway	(Joo et al., 2016; Martin et al., 2017)
Glycyrrhizin (triterpenes)	*Glycyrrhiza glabra* (Fabaceae)	TxA2 and JAK/STAT signaling pathway	(Deng et al., 2017)
Hispidulin (flavone)	*Salvia involucrate* (Lamiaceae)	Intrinsic apoptosis pathway	(Gao et al., 2017; Han et al., 2018)
HS-1793 (stilbenoid)	*Polygonum cuspidatum* (Polygonaceae)	HIF-1α, VEGF, Ki-67 and CD31	(Kim et al., 2017)
Licochalcone A (chalcone)	*Glycyrrhiza glabra* (Fabaceae)	Cyclins and CDKs	(Lu et al., 2018b)
Nimbolide (triterpene)	*Azadirachta indica* (Meliaceae)	PI3K/AKT/mTOR and ERK signaling	(Subramani et al., 2016)
Physapubescin B (Steroid)	*Physalis pubescens* L. (Solanaceae)	Ki-67, Cdc25C, and PARP	(Ding et al., 2015)
Pterostilbene (polyphenol)	*Polygonum cuspidatum* (Polygonaceae)	Mitochondrial mediated apoptosis; ERK and STAT3 signaling	(Feng et al., 2016; Kong et al., 2016; Wen et al., 2017)
Resveratrol (phenol)	*Polygonum cuspidatum* (Polygonaceae)	Regulating cell cycle and apoptosis pathways	(Banerjee et al., 2002)
Sulforaphane (organosulfur)	*Brassica oleracea* (Brassicaceae)	Cell cycle arrest and apoptosis. Targets: caspase 8, p21, hsp90	(Qazi et al., 2010)
Thymol (monoterpenoids)	*Thymus vulgaris* (Lamiaceae)	Mitochondrial mediated apoptosis	(De La Chapa et al., 2018)
Thymoquinone (quinone)	*Nigella sativa* (Ranunculaceae)	STAT3 and associated protein	(Zhu et al., 2016; Odeh et al., 2018)
Ursolic acid (triterpenoids)	*Oldenlandia diffusa* (Rubiaceae)	Ki-67, CD31, and miR-29a	(Prasad et al., 2012; Zhang et al., 2018)
Withaferin-A (phytosterols)	*Withania somnifera* (Solanaceae)	AKT signaling FOX03a-Par-4 cell death pathway, ERK, and p38 pathway	(Choi and Kim, 2015; Suman et al., 2016; Kuppusamy et al., 2017)

6-Shogaol is a minor, bioactive component isolated from ginger (*Zingiber officinale,* Roscoe). In a nude mice model of non-small cell lung cancer (NSCLC), 6-shogaol (10 mg/kg) significantly inhibited the growth of NCI-H1650 lung cancer cells which was associated with decreased cell proliferation and increased apoptosis as evidenced by reduced Ki-67-positive cells and an increased number of terminal deoxynucleotidyl transferase deoxyuridine triphosphate nick-end labeling (TUNEL)-positive cells. At *in vitro* level 6-shogaol suppressed Akt signaling through direct targeting of Akt1 and Akt2 (Kim et al., 2014). In a syngeneic FVB/N mice model of prostate cancer, intraperitoneal administration of 6-shogaol (100 mg/kg body weight) reduced tumor weight which was associated with decrease in pSTAT3Y705 and both cyclin D1 and survivin levels (Saha et al., 2014).

Allicin, one of the main organic allyl sulfur components in garlic (*Allium sativum,* Amaryllidaceae), was examined for its effects on cholangiocarcinoma (CCA) (Chen et al., 2018). In BALB/c nude mice model of CCA, allicin (10 mg/kg) significantly suppressed the growth of human liver bile duct carcinoma (HuCCT-1). The *in vitro* molecular study showed that allicin (20 µM) reduced the levels of matrix metalloproteinase (MMP)-2 and -9, *via* reducing the activity of the STAT3 signaling pathway to decrease migration, invasion, and epithelial-mesenchymal transition (EMT) of HuCCT-1 cell. Additionally, allicin suppressed proliferation by activating the caspase cascade, inducing apoptosis, and reducing the expression of proteins downstream of STAT3, such as B-cell lymphoma 2 (Bcl-2), while upregulating Bcl-2-associated X (Bax) protein (Chen et al., 2018). Subsequent studies, showed that allicin (5 µM) altered TIMP/MMP balance, *via* reducing the activity of the PI3K/AKT signaling pathway thereby significantly inhibiting adhesion, invasion, and migration of lung adenocarcinoma A549 and H1299 cells (Huang et al., 2017).

Alpinumisoflavone (AIF) is a pyranoisoflavone found in *Derris eriocarpa* (Leguminosae) plant. In BALB/c nude mice xenograft with human clear cell renal cell carcinoma (ccRCC) cell xenografts, AIF (40 mg/kg) suppressed growth, and metastasis of 786-O human ccRCC cells. The inhibitory effect was due to increase expression of miR-101 by suppressing Akt signaling (i.e., decreasing RLIP76 expression and p-Akt/t-Akt ratio) (Wang et al., 2017). In addition, AIF was reported to increase radiosensitivity in esophageal squamous cell carcinoma (ESCC) by suppressing the expression of nuclear transcription factor Nrf2 and Nrf2-driven antioxidant molecule NQO-1 and HO-1, aggravating reactive oxygen species (ROS) generation, DNA damage apoptosis, and cell cycle arrest (Zhang et al., 2017a).

Andrographolide is a bicyclic diterpenoid lactone isolated from *Andrographis paniculata* (Acanthaceae). Andrographolide was found to inhibit tumor growth by blocking tumor adaptation to hypoxic condition (Li et al., 2015). The observed effect of andrographolide (100 mg/kg) was due to inhibition of hypoxia-inducible factor (HIF)-1α activity and its upstream PI3k/AKT/mTOR pathway (Li et al., 2015). More details on the therapeutic potential of Andrographolide in cancer have been reviewed in Islam et al. (Islam et al., 2018).

Apigenin (APG), is a naturally occurring flavonoid present in fruits and vegetables with diverse anticancer properties (reviewed in Madunić et al., 2018). In athymic nude mouse xenograft with human chondrosarcoma Sw1353 cells, APG (5 mg/kg) suppressed tumor growth which was associated with decrease in Ki67 expression and induction of apoptosis (Yan et al., 2018). At molecular level APG regulated the expression of Bcl-2 family protein and activated the caspase cascade to induce G2/M phase arrest and apoptosis (Yan et al., 2018). In another study, APG (3 mg/kg) targeted dipeptidyl peptidase IV (DPPIV) enzyme to reduce the growth and metastasis of NSCLC xenografts. *In vitro* mechanistic investigations showed that APG suppressed the snail/slug signaling and downregulated DPPIV enzyme to modulate the EMT and the invasive ability of both EGFR positive and negative NSCLC cells (Chang et al., 2018). Some of preclinical studies showed that the efficacy of APG enhanced when combined with other chemotherapeutic agents (Hu et al., 2018) or loaded in nanocarriers (Bhattacharya et al., 2018).

Baicalein and baicalin are the naturally occurring flavonoids and active components of *Scutellaria baicalensis* (Lamiaceae). In NOD-scid IL2Rγ null (NSG) mouse xenograft with human colon cancer HCT116 cells, baicalein (50 mg/kg) and baicalin (50 mg/kg) inhibited tumor growth and induced apoptosis (Dou et al., 2018). At the *in vivo* level it down-regulated human telomerase reverse transcriptase (hTERT) expression, and deactivated mitogen-activated protein kinase (MAPK), extracellular receptor kinase (ERK), and p38 signaling pathways (Dou et al., 2018). In another study in nude mouse model of colon cancer, intraperitoneal administration of baicalin (50 mg/kg) inhibited tumor growth by repressing the expression of c-Myc and oncomiRs microRNAs to induce apoptosis (Tao et al., 2018). Furthermore, in combination with docetaxel (10 mg/kg), baicalein (50 mg/kg) additively inhibited the tumor growth by increasing apoptosis and decreasing tumor angiogenesis (Lu et al., 2018a).

Curcumin (phytopolylphenol) is a phytochemical from *Curcuma longa* (Zingiberaceae). Several studies have reported anticancer potential of curcumin through modulation of multiple signaling and gene expression regulatory pathways (Kunnumakkara et al., 2017). Curcumin inhibited the tumor growth in mice subcutaneously injected with human A375 melanoma cells. Studies indicated that curcumin inhibited the growth of melanoma cells through mechanisms including cell cycle arrest, autophagy, and downregulation of the PI3K/AKT/mTOR/P70S6K pathway which is a critical intracellular signaling pathway associated with cell survival and death (Zhao et al., 2016).

Decursin and decursinol are coumarins purified from the dried roots of *Angelica gigas* Nakai. Decursin is rapidly and extensively converted to decursinol in rodents and humans (Zhang et al., 2015; Wu et al., 2017). In SCID-NSG mice xenograft with human prostate cancer LNCaP/AR-Luc cells overexpressing the wild type androgen receptors (AR), decursinol (4.5 mg/mouse) decreases tumor growth and lung metastasis (Wu et al., 2017).

Dicumarol (DIC) is the natural anticoagulant derived from coumarin, by bacterial action in spoiled sweet clover hay (*Melilotus officinalis*, Fabaceae). In BALB/c nude mouse xenograft model, DIC (30 mg/kg) significantly suppressed the growth of SKOV3 ovarian carcinoma cells (Zhang et al., 2017b). The *in vitro* molecular mechanistic studies suggested that DIC inhibited the kinase activity of pyruvate dehydrogenase kinase 1 (PDK1), shifted the glucose metabolism from aerobic glycolysis to oxidative phosphorylation, generated a higher level of ROS, attenuated the mitochondrial membrane potential (MMP), induced apoptosis, and reduced cell viability of SKOV3 cells. Notably, DIC (32 mg/kg) was found safe toward ovarian tissues and developing oocytes; implicating importance of DIC as a potential anticancer agent when female fertility preservation is a concern (Aras et al., 2016).

Epigallocatechin (EGCG), a major catechin found in green tea, effectively delayed the tumor incidence and reduced tumor burden by inducing apoptosis and inhibiting proliferation of human breast cancer MDA-MB-231 cells in nude mouse model (Thangapazham et al., 2007). In another study, EGCG suppressed the increase of oxidative stress-derived DNA damage marker 8-hydroxydeoxyguanosine (8-OH-dGuo) levels in mouse lung DNA to inhibit nitrosamine (NNK)-induced lung tumorigenesis (Xu et al., 1992).

Emodin is an anthraquinone derivative from the root and rhizome of *Rheum palmatum* L. (Polygonaceae). In a BALB/c nude mice, emodin (50 mg/kg) inhibited the growth of human lung epithelial (A549) cells by inducing endoplasmic reticulum (ER) stress-dependent apoptosis. The *in vitro* molecular mechanism showed that emodin activated ER stress and TRIB3/nuclear factor-κB signaling (Su et al., 2017). In mice bearing EO771 or 4T1 breast tumors, emodin suppressed tumor growth by inhibiting macrophage infiltration and M2-like polarization, accompanied by increased T-cell activation and reduced tumor angiogenesis (Iwanowycz et al., 2016). At molecular level, emodin inhibited IRF4, STAT6, and C/EBPβ signaling and significantly increased inhibitory histone H3 lysine 27 tri-methylation (H3K27m3) on the promoters of M2-related genes in tumor-associated macrophages (Iwanowycz et al., 2016). In BALB/c nude mice xenograft with human hepatocellular cancer SMMC-7721 cells, emodin suppressed tumor growth and induced apoptosis with increases in ERK and p38 phosphorylation and suppression of p-JNK expression (Lin et al., 2016).

Genistein is a naturally occurring isoflavone present in soy beans with estrogen-like properties. Genistein (140 mg/kg) treatment decreased the number of total aberrant crypts in the azoxymethane (AOM)-induced rat colon cancer model through the inhibition of aberrant nuclear accumulation of β-catenin and suppression of WNT signaling genes (Zhang et al., 2013). In athymic BALB/c nu/nu mouse xenograft with human leukemia cell line HL‐60, intraperitoneal injected of genistein (0.4 mg/kg) for 28 days significantly reduced the tumor weight without affecting the body weight (Hsiao et al., 2019). At *in vitro* level, genistein‐induced G2/M phase arrest and apoptosis of HL‐60 cells through ROS mediated ER stress leading to increased Ca2+ production and decreased mitochondrial membrane potential. At molecular level, the observed effect was due to increased expression of ER stress-associated proteins (IRE‐1α, calpain 1, GRP78, GADD153, caspase‐7, caspase‐4, and ATF‐6α) and apoptosis associated proteins (Bax, PARP‐cleavage, caspase‐9, caspase‐3, Bcl‐2, and Bid) (Hsiao et al., 2019).

Gingerol is a major phenolic compound present in the rhizomes of ginger (*Z. officinale* Roscoe). In a syngenic mouse model of spontaneous breast cancer metastasis, gingerol (5 mg/kg) treatment induced caspase-3 activation and inhibited the orthotopic tumor growth as well as metastasis of mouse brain-metastatic 4T1Br4 mammary tumor cells to multiple organs such as lung, bone and brain (Martin et al., 2017). Likewise, Joo and colleagues (Joo et al., 2016) reported inhibition of lung-metastatic, MDA-MB-231 human breast cancer cell proliferation, and invasion by [10]-gingerol through suppression of Akt, p38MAPK, and epidermal growth factor receptor. The detailed protective and therapeutic potential of gingerol in cancer is reviewed in de Lima et al. (2018).

Glycyrrhizin (GA) is the major bioactive component found in licorice roots of a small leguminous shrub, *Glycyrrhiza glabra* L. In athymic BALB/c nude mice xenograft with human lung adenocarcinoma A549 cells stably transfected with TxA2 receptor (TPα), GA (135 mg/kg) reduced thromboxane synthase (TxAS) and proliferating cell nuclear antigen (PCNA) expression *via* suppressing TxA2 pathway (Deng et al., 2017). More recent findings showed that GA (100 mg/kg) inhibited the growth of non-small cell lung cancer cells (NSCLC) in patient-derived xenograft (PDX) mice by suppressing the level of high mobility group box 1 (HMGB1) and inhibition of JAK/STAT signaling pathway (Wu et al., 2018b).

Hispidulin is a phenolic flavonoid compound found in different plant materials such as *Saussurea involucrata* Kar (Asteraceae). Intraperitoneal administration of hispidulin (20 mg/kg) inhibited the Caki-2 (human clear cell renal cell carcinoma) tumor growth and lung metastasis in athymic BALB/c nu/nu mouse model by increasing the expression of cleaved caspase-3 and decreasing the activity of Sphk1, thereby modulating ceramide-S1P balance (Gao et al., 2017). Similarly, in another study, hispidulin (20 mg/kg) effectively suppressed human hepatocellular carcinoma Bel7402 cell xenograft tumor growth and lung metastasis in by increasing the expression of PPARγ and phosphorylation levels of AMPK, JNK and ERK proteins (Han et al., 2018).

HS-1793 is a synthetic analogue of resveratrol with improved photosensitivity and stability profile. In a nude mouse model of breast cancer, HS-1793 (5 mg/kg) significantly suppressed the growth of human breast cancer MDA-MB-231 cells with decreased expression of Ki-67 and CD31 proteins. Moreover, HS-1793 treatment downregulated expression of HIF-1 and vascular endothelial growth factor (VEGF) protein both of which are key components of the angiogenic process (Kim et al., 2017). Apart from its growth inhibitory and antiangiogenesis effects, HS-1793 enhanced ionizing radiation-induced apoptosis and inhibited hypoxia-induced cancer stem cell properties in hypoxic mouse breast cancer FM3A cells (Choi et al., 2016).

Licochalcone A (LicA) is a phenol chalconoid isolated from the roots of *Glycyrrhiza* species. In athymic BALB/c nu/nu mouse model, LicA (20 mg/kg) inhibited the human cervical cancer cell SiHa tumor growth *via* inhibition of the PI3K/Akt/mTOR signaling pathway and induction of apoptosis (Tsai et al., 2015). In athymic nude mice subcutaneous or orthotopic xenograft with human glioma U87 cells, LicA induced cell cycle arrest in the G0/G1 and G2/M phases by reducing the expression of cyclins and cyclin-dependent kinases (Lu et al., 2018b). Most recently, LicA was shown to suppress hexokinase 2-mediated tumor glycolysis in gastric cancer *via* downregulation of the Akt signaling pathway (Wu et al., 2018a).

Nimbolide is a triterpene derived from the leaves and flowers of the neem tree (*Azadirachta indica*). In an athymic nu/nu mouse model, nimbolide (5 mg/kg) inhibited the pancreatic cancer HPAC cell growth and metastasis by inducing apoptosis (Subramani et al., 2016). The *in vitro* molecular mechanism studies showed that nimbolide increased ROS generation, inhibited proliferation (through reduced PI3K/AKT/mTOR and ERK signaling) and metastasis (through decreased EMT, invasion, migration, and colony forming abilities) *via* mitochondrial-mediated apoptotic cell death. Recent *in vitro* study suggested epigenetic role of nimbolide in regulating autophagy and apoptosis in human breast cancer cells (Pooladanda et al., 2018).

Physapubescin B is a steroidal substance isolated from *Physalis pubescens* L. (Solanaceae). In nude mouse models with prostate cancer xenografts, physapubescin B (50 mg/kg) decreased PC3 tumor growth by reducing the expression levels of Ki-67, Cdc25C, and full length PARP and increasing the apoptotic cell population within the tumor tissue (Ding et al., 2015). Furthermore, in renal cell carcinoma 786-O cells, physapubescin (30 mg/kg) decreased the protein expression of vimentin and inhibited *in vivo* angiogenesis (Chen et al., 2016).

Pterostilbene is a naturally occurring derivative of resveratrol originated from grape (*Vitis vinifera, Vitaceae*). In an athymic nude mouse esophageal cancer model, pterostibene (100 or 200 mg/kg) significantly inhibited EC109 tumor growth, cell adhesion, migration, and intracellular glutathione (GSH) levels while increasing the apoptotic index, caspase 3 activity, and ROS levels (Feng et al., 2016). Similarly, in a athymic nude mouse model of diffuse large B-cell lymphoma, pterostibene (30 mg/kg) markedly inhibited tumor growth, reduced MMP, increased cellular apoptotic index and ROS levels, leading to S-phase arrest in the cell cycle (Kong et al., 2016). More importantly, it was demonstrated that pterostilbene (30 mg/kg) with megestrol acetate (10 mg/kg) significantly reduced HEC-1A tumor growth in an endometrial cancer xenograft mouse model as compared to pterostilbene or megestrol acetate alone (Wen et al., 2017). At *in vitro* level, the above combination suppressed ERK and STAT3 signaling pathways and estrogen receptor expression.

Resveratrol is a polyphenolic phytoalexin (stilbenoid). Numerous reports have shown that resveratrol suppresses proliferation of a wide variety of tumor cells, including breast, colon, prostate, liver, and lung (Banerjee et al., 2002). Resveratrol significantly reduced tumor growth and metastasis to the lung in mice bearing highly metastatic Lewis lung carcinoma tumors (Kimura and Okuda, 2001). The results suggested that the antitumor and antimetastatic activities of resveratrol could result from the inhibition of DNA synthesis, inhibition of neovascularization, and angiogenesis. In 7,12-dimethylbenz(a)-anthracene (DMBA)-induced mammary cancer model, resveratrol reduced the incidence and multiplicity of tumors, concurrently extending the latency period. In the same study, resveratrol could suppress activation of nuclear factor-κB which regulates the gene expression of cyclooxygenase-2 and matrix metalloproteinase-9 (Banerjee et al., 2002).

Sulforaphane (SFN) is a compound within the isothiocyanate group of organosulfur compounds. SFN exerts its anticancer effects by modulating key signaling pathways such as induction of apoptosis, inhibition of cell cycle progression, inhibition of angiogenesis, and by increasing anticancer activity of other antiproliferative agents including paclitexal (Qazi et al., 2010; (Su et al., 2018). Addition of SFN and paclitexal to Barrett esophageal adenocarcinoma (BEAC) cells significantly increased apoptotic cell death compared to SFN or paclitexal (Qazi et al., 2010). A significant reduction in tumor volume was also observed by SFN in severe combined immunodeficient (SCID) mice subcutaneously injected with BEAC cells (Qazi et al., 2010).

Thymol is a transient receptor potential ankyrin subtype 1 (TRPA1) channel, agonist found in thyme (*Thymus vulgaris*) and oregano (*Origanum vulgare*). In oral squamous cell carcinoma Cal27‐ and HeLa‐derived mouse xenografts, intratumor injection of thymol (4.3 mmol/L) reduced the tumor volume with decreasing cell proliferation and inducing apoptosis as observed by Ki-67 staining and TUNEL assays, respectively (De La Chapa et al., 2018). The *in vitro* molecular mechanism studies showed that thymol induced depolarization of mitochondrial membrane potential to induce apoptosis (De La Chapa et al., 2018).

Thymoquinone (2-isopropyl-5-methyl-1,4-benzo-quinone, TQ) is the active constituent of black cumin (*Nigella sativa, Ranunculaceae*) seed oil. In a BALB/c athymic nude mice, TQ (10 mg/kg) decreased tumor weight and size by inducing apoptosis and inhibiting STAT3 phosphorylation in human gastric cancer cells. The downregulation of STAT3 activation was associated with a reduction in JAK2 and c-Src activity (Zhu et al., 2016). Recent preclinical studies suggested the potential of TQ in adjuvant therapy with other chemotherapeutic agents (reviewed in Mostofa et al., 2017). In another study in BALB/c mice transplanted with mouse epithelia breast cancer EMT6/P cell line, TQ in combination with melatonin significantly decreased the tumor size, induced tumor cell death, decreased VEGF expression, and activated anticancer immune response by increasing serum interferon (INF)-γ level (Odeh et al., 2018).

Ursolic acid (UA) is a natural terpene compound found in a variety of natural plants. Anticancer activity of UA is well known with recent studies suggesting the use of UA as a cancer chemosensitizer to standard chemotherapeutic drugs (Prasad et al., 2016). In one study UA was shown to enhance the therapeutic effects of oxaliplatin in mouse model of CRC by inhibiting the tumor and increasing the survival rate. The *in vitro* mechanistic study suggested that treatment of CRC cells with UA and oxaliplatin significantly inhibited cell proliferation, increased apoptosis and ROS production, and significantly inhibited expression of drug resistant gene (Zhang et al., 2018). The UA nanoparticles decreased tumor size by targeting caspases and p53 with downregulation of Bcl-2 and cIAP, inducing apoptosis and leading to cervical cancer cell death (Wang et al., 2018).

Withaferin A (WA) is a steroidal lactone present in *Withania somnifera* (Solanaceae). In a nude mouse model of colorectal cells (CRC), oral administration of WA (5 mg/kg) inhibited the tumor growth of human colorectal carcinoma (HCT-116) cells overexpressing AKT and micro-vessel formation. At *in vitro* level in AKT overexpressing HCT-116 cells, WA inhibited cell proliferation, migration, and invasion by downregulating EMT markers (snail, slug, β-catenin, and vimentin) (Suman et al., 2016). In another study, intraperitoneal administration of withaferin-A (2 mg/kg) inhibited CRC growth by blocking interleukin-6-induced activation of STAT3 (Choi and Kim, 2015). Similarly, in another study, oral administration of WA (4 mg/kg) effectively inhibited HepG2-xenografts and diethylnitrosamine (DEN)-induced-hepatocellular carcinoma (HCC) in C57BL/6 mice by elevating the levels of ERK, RSK, ELK1, and DR5 along with decreased expression of Ki67. The *in vitro* molecular mechanism studies suggested WA increased phosphorylation of ERK and p38 leading to increased phosphorylation of p90-ribosomal S6 kinase (RSK) and a concomitant activation of ETS-like transcription factor-1 (ELK1) and death receptor protein-5 (DR5) (Kuppusamy et al., 2017).

## Phytochemicals Evaluated in Clinical Trials

**Figure 2:**
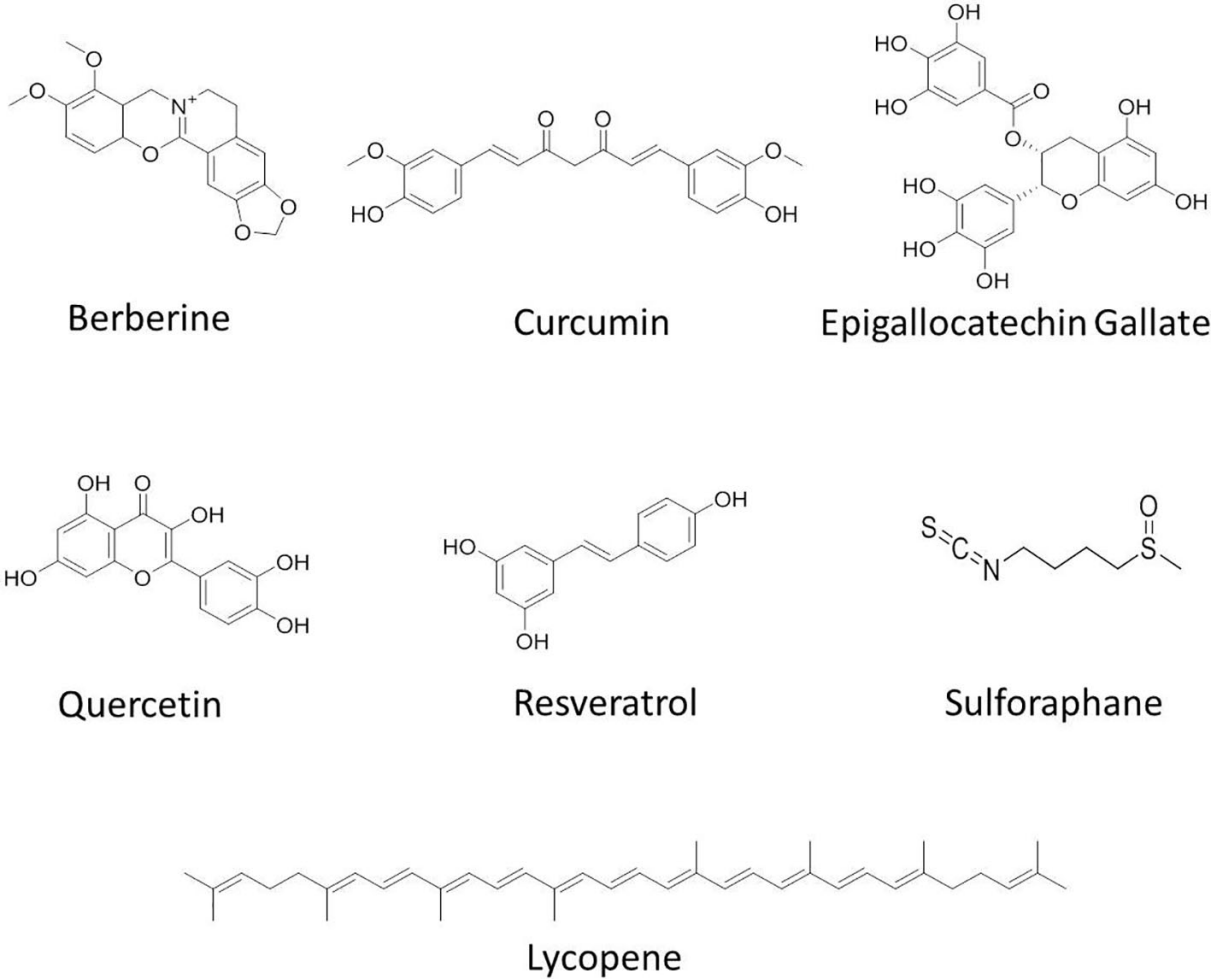
Chemical structures of some anticancer agents in clinical trials.

Clinical trials using phytochemicals against cancer are still in infancy through an overwhelming large number of anti-cancer compounds are currently under development. The clinical trials with phytochemicals focus on three major aspects of cancer research: 1) improving the response of cancer cells toward standard chemo- and radiotherapy, 2) reducing the severe adverse effects of standard cancer therapy, and 3) looking for unwanted interactions with standard therapy. Preclinical studies have shown the effectiveness of various phytochemicals such as berberine, curcumin, green tea, catechins including EGCG, lycopene, quercetin, resveratrol, and sulforaphane (**Figure 2**). The phytochemicals which are currently under clinical trials against various cancers are summarized in **Table 3** and their brief description is given below:

**Table 3:** List of phytochemicals currently in clinical trial on various cancers.

Phytochemical	Class of compound	Type of cancer	Assessment	Reference
Berberine (alkaloid)	*Berberis* sp. (Berberidaceae)	Colorectal cancer	Prevention of recurrence	NCT03281096*
Curcumin (polylphenol)	*Curcuma longa* (Zingiberaceae)	Advanced and metastatic breast cancer	Quality of life, safety in combination, progression free survival, time to disease Progression, and time to treatment failure	NCT03072992*
Epigallocatechin (flavonoids)	*Camellia sinensis* (Theaceae)	Colorectal cancer	Change in methylation pattern compare to baseline	NCT02891538*
Lycopene (carotenoids)	*Solanum lycopersicum* (Solanaceae)	Metastatic colorectal cancer	Effectiveness in reducing skin toxicity alone or in combination with panitumumab. Pharmacokinetics.	NCT03167268*
Quercetin (carotenoids)		Prostate cancer	EGCG, ECG, quercetin, and their methylated metabolites in prostate tissue and plasma. Enzyme activity expression of COMT, DNMT1, and MRP1. Inter-individual variation in genotype of COMT	NCT01912820*
Resveratrol (stilbenoid)	*Polygonum cuspidatum* (Polygonaceae)	Low-grade GI neuroendocrine tumors	Notch1 activation, toxicity	NCT01476592*
Sulforaphane (isothiocyanate)	*Brassica oleracea* (Brassicaceae)	Former smokers with a high risk of developing lung cancer	Bronchial dysplasia index, cell proliferation marker Ki-67, apoptosis markers including caspase-3 and TUNEL	NCT03232138*

*Indicates reference found at www.clinicaltrials.gov with corresponding identifier code (NCT).

Berberine, a benzyl-tetra isoquinoline alkaloid found in *Berberis* sp. (Berberidaceae) has long been a part of traditional Chinese and Ayurvedic medicine. Preclinical efficacy of berberine has been established in various cancers including colon (Mao et al., 2018), breast (Zhao et al., 2017), gastrointestinal (Hesari et al., 2018), oral (Lin et al., 2017), liver (Tsang et al., 2015), pancreas (Abrams et al., 2019), prostate (Youn et al., 2018), ovarian (Hou et al., 2017), and cervical (Mahata et al., 2011) cancers. Despite large preclinical efficacy data, clinical trials related to the evaluation of true potential of berberine as an anticancer agent are limited. Most of the clinical trials have demonstrated the safety of berberine against other clinical conditions such as type 2 diabetes. In a randomized, double-blind and placebo-controlled phase 3 clinical trial, administration of berberine (1 g/day) was found to be safe in type 2 diabetic patients with dyslipidemia (Zhang et al., 2008). Currently, a randomized, double-blind, placebo-controlled phase 2/3 trial is ongoing to determine the efficacy of berberine hydrochloride (300 mg/twice/day) against the occurrence of new colorectal adenomas among 1,000 patients with a history of colorectal cancer (NCT03281096).

Curcumin, a yellow polyphenolic pigment, is an active ingredient in turmeric (*Curcuma longa;* Zingiberaceae) and is a highly promising chemopreventive agent. Several groups reported the chemopreventive and chemotherapeutic role of curcumin in different cancer cells including blood (Taverna et al., 2015), breast (Mock et al., 2015), head and neck (Wilken et al., 2011), liver (Darvesh et al., 2012), prostate (Nakamura et al., 2002), ovary (Yallapu et al., 2010), and skin cancers (Huang et al., 1997). This has warranted studies in clinical trials to address pharmacokinetics, safety, and efficacy issues of curcumin in humans. Phase I clinical trials have shown safety, tolerability, and nontoxicity of curcumin even at high doses (8 g/day) but exhibited poor bioavailability in humans (Sharma et al., 2004; Kanai et al., 2013). Despite bioavailability challenges, clinical trials with curcumin either alone or in combination as an anticancer agent have shown efficacy against several disease sites such as breast (Bayet-Robert et al., 2010), prostate (Mahammedi et al., 2016), pancreatic (Epelbaum et al., 2010; Kanai et al., 2013), colorectal (Sharma et al., 2004; Carroll et al., 2011; Irving et al., 2015; James et al., 2015), and hematological malignancies (Ghalaut et al., 2012). Latest information on various preclinical and clinical anticancer trials using curcumin is reviewed in Doello et al. (2018). Recently, in patients with locally advanced or metastatic pancreatic cancer, curcumin Meriva^®^ (2,000 mg/day) in complementary to gemcitabine was found to increase the efficacy of gemcitabine without any treatment-related toxicity (Pastorelli et al., 2018). Currently, a randomized, double-blind, placebo-controlled phase 2/3 trial is ongoing to determine the efficacy of curcumin (300 mg/i.v./day) along with Paclitaxel (80 mg/m2 BS; i.v.) administrated once weekly for 12 weeks against the advanced and metastatic breast cancer patients (NCT03072992). Apart from this study, 18 other actively ongoing oncology-based trials using curcumin are registered on clinicaltrials.gov.

 Epigallocatechin (EGCG) is a major catechin found in green tea (*Camellia sinensis;* Theaceae). Numerous studies using cell lines and animal models have established anticancer activity of EGCG (Wang and Bachrach, 2002; Fujiki et al., 2015). Data from clinical trials provide evidence of safety of catechin mixture containing EGCG (200 mg/day) in men diagnosed with high-grade prostatic intraepithelial neoplasia (HGPIN) and/or atypical small acinar proliferation (ASAP) (Kumar et al., 2016). In a randomized, presurgical placebo-controlled phase II pilot study of polyphenon E (a green tea polyphenol formulation primarily consisting of EGCG; 1,200 mg/day) in bladder cancer patients, EGCG accumulated in cancer tissue and decreased the level of proliferation (PCNA) and apoptosis (clusterin) biomarkers (Gee et al., 2017). Moreover, recent study has suggested the use of EGCG in combination with indole-3-carbinol for better treatment outcomes in advanced ovarian cancer patients (Kiselev et al., 2018). Currently, a randomized, early phase 1 trial is ongoing to evaluate the chemopreventive effects of Teavigo™ (highly purified and refined green tea extract providing 94% EGCG) (450 mg/PO/day) in colorectal cancer (CRC) patients with curative resections (NCT03072992).

Lycopene, a naturally occurring chemical that gives fruits and vegetables a red color, is abundantly found in red tomatoes (*Solanum lycopersicum;* Solanaceae). In the meta-analysis of Chen et al. (6 cohort and 11 nested case-control studies), the intake of lycopene/tomato was associated with relatively minor reduction in the risk of prostate cancer diagnosis in men consuming a higher level of lycopene (Chen et al., 2013). In a randomized, double blinded, controlled trial in patients with multifocal high grade prostatic intraepithelial neoplasia (HGPIN) and/or atypical small acinar proliferation (ASAP), administration of high dose supplement containing lycopene (35 mg), selenium (55 µg), and 600 mg green tea catechins (GTCs) for 6 months, insignificantly decreased the prostate specific antigen (PSA) levels, but increased incidence of prostate cancer at re-biopsy and expression of microRNAs associated with prostate cancer progression (Gontero et al., 2015). The study suggested avoiding the use of high doses of supplements in patients with prostatic intraepithelial neoplasia (Gontero et al., 2015). Interestingly, in a recent metabolomic study on men with increased PSA levels but no prostate cancer, intake of lycopene (15 mg) along with GTCs (EGCG 600 mg) for 6-months reduced the levels of circulating pyruvate. The study using Mendelian randomization analysis suggested association of pyruvate level with prostate cancer risk (Beynon et al., 2019). Overall, with the scarcity and heterogeneity of existing clinical evidences, the conclusions drawn can be conflicting or ambiguous. Nevertheless, currently double-blind, placebo-controlled phase 2 trial is ongoing to assess the effectiveness of lycopene (20 mg/PO/day) to reduce skin toxicity in metastasis colorectal carcinoma patients treated with panitumumab (NCT03167268).

Resveratrol (3,5,4′-trihydroxy-trans-stilbene) is a stilbenoid, which is found largely in the skins of red grapes (*Polygonum cuspidatum*; Polygonaceae). In a phase I study on men with elevated PSA level in recurrent prostate cancer, pulverized muscadine grape skin extract (MPX) containing 4,000 mg resveratrol, compared with placebo, delayed the development of recurrence by lengthening the prostate specific antigen doubling time (PSADT) by 5.3 months (Paller et al., 2015). Moreover, 12-month treatment with MPX did not significantly prolong PSADT over two different doses, low (500 mg) or high (4,000 mg) (Paller et al., 2018). In a pilot study on patients with colorectal cancer with hepatic metastases, resveratrol (5.0 g/day for 14 days) was detected in hepatic tissue where cleaved caspase 3, a marker of apoptosis, was significantly increased in malignant hepatic tissue (Howells et al., 2011). In another pilot study on 39 women at increased risk for breast cancer, trans-resveratrol (50 mg twice a day for 12 weeks) decreased methylation of Ras association domain family 1 isoform A (RASSF)-1a, a gene associated with breast cancer, increased levels of trans-resveratrol and resveratrol-glucuronide in the circulation, and decreased cancer promoting PGE2 expression in the breast (Zhu et al., 2012). Recently, a clinical trial aimed at studying the effect of resveratrol (2.5 gm/p.o./twice/day) on Notch-1 signaling in low-grade gastrointestinal neuroendocrine tumors was completed (NCT01476592). However No study results are posted so far on this clinical trial.

Sulforaphane (SFN) is a dietary isothiocyanate found in cruciferous plants such as broccoli (*Brassica oleracea, Brassicaceae*). Cipolla et al. conducted a double-blinded, randomized, placebo-controlled trial with SFN in 78 patients with increased PSA levels after radical prostatectomy. Oral administration of sulforaphane (60 mg/day) for 6 months significantly increased PSA doubling time (PSADT) and did not show any adverse events as compared to the placebo group. Moreover, PSA slopes which were measured 2 months after SFN treatment remained the same (Cipolla et al., 2015). In a single arm trial, Alumkal et al. carried out the efficacy, safety, pharmacokinetics, and pharmacodynamics study of SFN-rich broccoli sprout extracts (200 μmoles/day) administrated for 20 weeks in patients (20) with biochemical (PSA) recurrent prostate cancer. Even though, the primary endpoint was not achieved, there was a significant increase in on-treatment PSADT as compared to pre-treatment PSADT (6.1 months pre-treatment *vs.* 9.6 months) (Alumkal et al., 2015). Currently, double-blind, placebo-controlled phase 2 trial is ongoing to assess the chemopreventive effect of Avmacol (sulforaphane) tablets (120 µM/p.o./twice/day) in former smokers with a high risk of developing lung cancer (NCT03232138).

## Phytochemicals Used in Current Cancer Therapy

**Figure 3:**
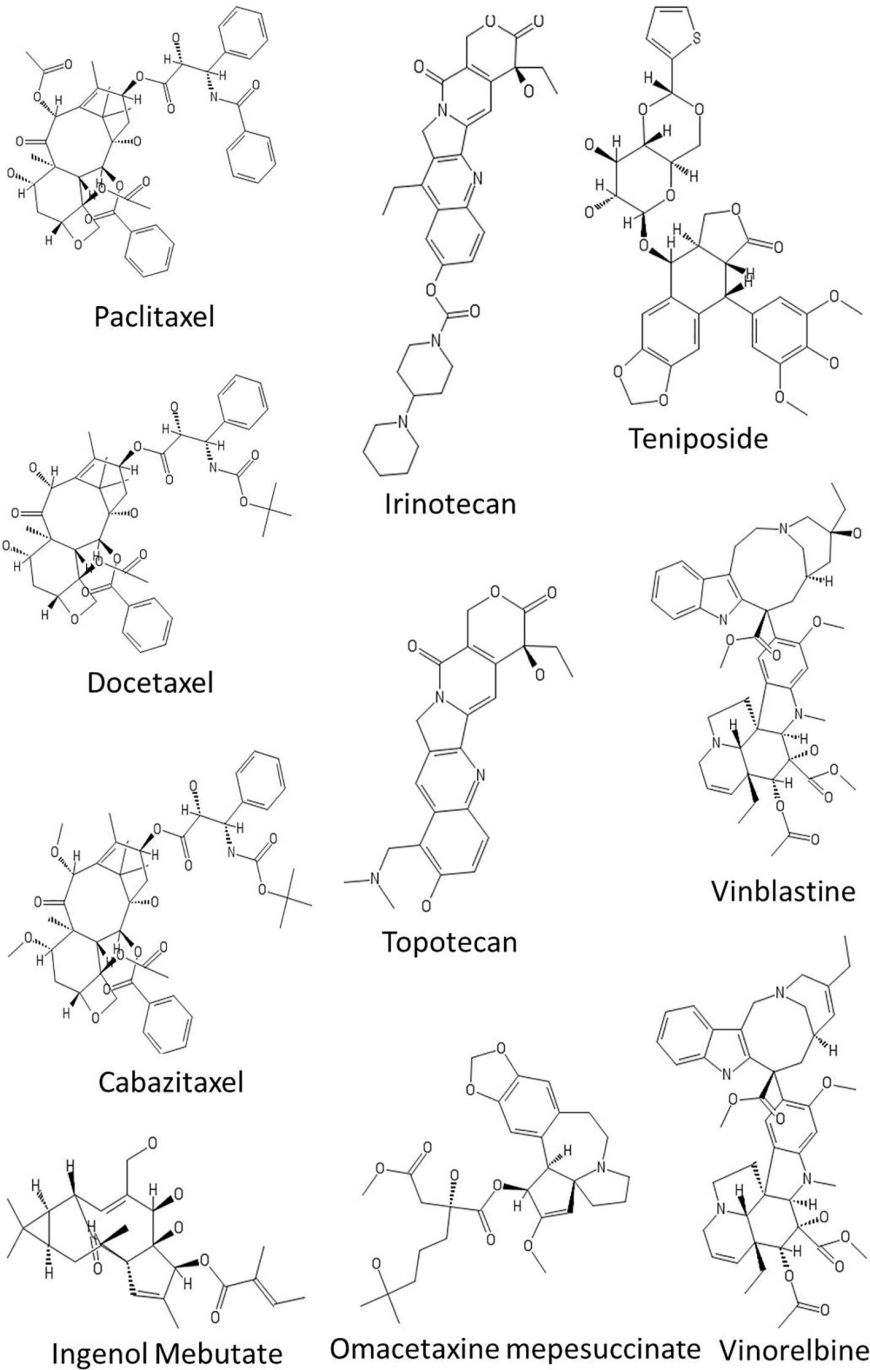
Chemical structures of some anticancer agents in clinical use.

The four major classes of clinically used plant-derived anticancer compounds include vinca alkaloids, taxane diterpenoids, camptothecin derivatives, and epipodophyllotoxin (**Figure 3** and **Table 4**). Apart from these phytochemical classes, other plant-derived anticancer agents from different classes such as combretastatins, homoharringtonine (omacetaxine mepesuccinate, cephalotaxine alkaloid), and ingenol mebutate are also used (**Figure 3** and **Table 4**). Poor aqueous solubility and significant toxic side effects still remain the major concern and therefore, the current focus of research is toward eradicating the impact of these factors. In this context, several analogues and prodrugs have been synthesized and methods have been devised to enhance aqueous solubility and tumor specificity. Brief description of a few phytochemicals which are used in cancer therapy is given below:

**Table 4:** Compounds used for cancer treatment.

Class of phytochemical	Pharmacological action	Type of cancer	Molecular targets	
**Vinca alkaloids**				
Vinblastine Vincristine Vindesine Vinflunine Vinorelbine	Inhibit microtubule polymerization and assembly, leading to metaphase arrest and cell death.	Non-small-cell lung carcinoma (NSCLC), breast, lung, leukemia, Hodgkin and non-Hodgkin lymphomas, testicular carcinoma, Kaposi’s sarcoma, and second-line transitional cell carcinoma of the urothelium (TCCU)	Tubulin	(Martino et al., 2018)
**Taxanes**				
Cabazitaxel Docetaxel Paclitaxel	Inhibit microtubule function resulting in cell cycle arrest and aberrant mitosis	NSCLC, head and neck, breast, prostate, gastric adenocarcinoma	Tubulin	(Kotsakis et al., 2016; Oudard et al., 2017)
**Podophyllotoxin**				
Etoposide Teniposide	Inhibits DNA synthesis by forming a complex with topoisomerase II and DNA.	Osteosarcoma, NSCLC cervical, nasopharyngeal, colon, breast, prostate, and testicular cancer	Topoisomerase II	(Cao et al., 2015)
**Camptothecin**				
Irinotecan Topotecan	Stabilizes topoisomerase I-DNA complex thereby preventing religation of single strand breaks resulting in lethal double-stranded breaks in DNA.	Ovarian, cervical, colorectal, and small cell lung cancer (SCLC)	Topoisomerase I	(Hertzberg et al., 1989)
**Other plant-derived anticancer agents**				
Combretastatin A4	Inhibits polymerization of tubulin causing disruption of the tumor endothelial cells lining the tumor vasculature	Polypoidal choroidal vasculopathy, anaplastic thyroid cancers	Tubulin	(Tozer et al., 2002)
Homoharringtonine	Binds to large ribosomal subunit, which affects chain elongation and prevents protein synthesis	Chronic myeloid leukemia	Ribosomoal protein	(Itokawa et al., 2005)
Ingenol mebutate	Rapid induction of cell death and activation of inflammatory response	Actinic keratosis	Protein kinase C	(Skroza et al., 2018)

### Vinca Alkaloids

Vinca alkaloids are a subset of drugs obtained from the pink periwinkle plant *Catharanthus roseus* (Apocynaceae). The Vinca alkaloids achieve cytotoxic effects by binding to β-tubulin at a site distinct from that of the taxanes thereby inhibiting polymerization and assembly of microtubules, leading to metaphase arrest and cell death. As the microtubules are associated with several other cellular functions such as maintenance of cell shape, motility, and transport between organelles, the vinca alkaloids affect both malignant and non-malignant cells in the non-mitotic cell cycle. Vinblastine and vincristine are the two naturally isolated alkaloids that have been used in clinical oncology for almost 50 years. A series of semisynthetic analogues of these two alkaloids have been developed (**Table 4**). Vinorelbine and vindesine are the two effective semisynthetic analogues that are approved for clinical use. These agents have been generally included in combination chemotherapy for the treatment of a variety of cancers, including leukemia, Hodgkin and non-Hodgkin lymphomas, advanced testicular carcinoma, breast and lung cancers, and Kaposi’s sarcoma. Recently, vinflunine, a second-generation gem-difluoromethylenated derivative of vinorelbine, has been approved for the treatment of second-line transitional cell carcinoma of the urothelium (TCCU). A comprehensive discussion of these agents is presented in the review by (Martino et al., 2018).

### Taxanes

Taxanes represent promising anticancer drugs that were first isolated from the bark of the Yew tree. Taxanes exert an anticancer affect by stabilization of microtubules, resulting in cell cycle arrest and aberrant mitosis. Paclitaxel, a natural product isolated from the bark and leaf of *Taxus brevifolia* and docetaxel, a semi synthetic derivative, is primarily used in breast, ovarian, pancreas, prostate, and lung cancer therapies. A number of semisynthetic derivatives have been developed with improved cytotoxicity in resistant tumors, decreased toxicity, and improved solubility. For example, cabazitaxel a second-generation docetaxel derivative exhibits cytotoxic activity against various docetaxel-resistant tumors with less overall toxicity (Kotsakis et al., 2016; Oudard et al., 2017). An additional characteristic of cabazitaxel is its ability to penetrate the blood–brain barrier *in vivo*, which is not achievable with other taxanes. Some of the paclitaxel analogues such as larotaxel, milataxel, ortataxel, and tesetaxel are currently undergoing clinical evaluation.

### Camptothecins

Camptothecin is a quinolone alkaloid isolated from the Chinese tree *Camptotheca acuminata*. Camptothecin complexes with type I DNA topoisomerase preventing both cleavage and religation of DNA leading to a DNA double-strand break and cytotoxicity (Hertzberg et al., 1989). At present, irinotecan and topotecan are the two FDA approved semi-synthetic camptothecin derivatives that are clinically active and less toxic than the parent compound. Irinotecan is prescribed for treatment of advanced cancers of the large intestine and rectum. Whereas, topotecan is approved for the treatment of recurring ovarian, small cell lung cancer, and cervical cancer.

### Podophyllotoxins

Podophyllotoxin is a natural product isolated from *Podophyllum peltatum* and *Podophyllum emodi (Berberidaceae).* Podophyllotoxin reversibly binds to tubulin, whereas its key derivatives etoposide and teniposide inhibit topoisomerase II, inducing topoisomerase II-mediated DNA cleavage. Moreover, podophyllotoxin also exhibits potential anti-multidrug resistant (MDR) activity against diverse drug-resistant tumor cells. For example, CIP-36, a podophyllotoxin derivative, has been shown to overcome the MDR of adriamycin-resistant human leukemic cell line K562/ADR by regulating the activity of topoisomerase-IIa (Cao et al., 2015). However, CIP-36 failed in clinical trials due to lack of efficacy and unacceptable toxicity.

### Other Plant-Derived Anticancer Agents

Ingenol mebutate (IM) is a hydrophobic ester of the diterpene ingenol isolated from common Australian plant *Euphorbia peplus* (Euphorbiaceae). IM is approved for the topical treatment of actinic keratosis, a common skin condition that results from exposure to chronic ultraviolet radiation which can lead to squamous cell carcinoma, if not treated. IM presents two mechanisms of action: at high concentrations (~200–300 µM), it induces rapid induction of cell death in the treated area and at low concentrations (~0.1 µM) it activates inflammatory response, capable of eliminating the residual cells. Pharmacology, mode of action, pharmacokinetics, dosing, and rout of administration of ingenol mebutate have been reviewed in more details by Skroza et al. (2018).

Homoharringtonine (HHT) is a naturally-occurring ester of the alkaloid cephalotaxine isolated from various trees of the *Cephalotaxus* genus (Cephalotaxaceae) and is approved for the treatment of chronic myeloid leukemia. HHT binds to the A-site cleft in the large ribosomal subunit, which affects chain elongation and prevents protein synthesis. The discovery and development of HHT and related compounds is comprehensively reviewed by Itokawa et al. (Itokawa et al., 2005). A semi-synthetic version of HHT, also known as omacetaxine mepesuccinate, has been reported to be an effective treatment for myelodysplastic syndromes (MDS) and chronic myelomonocytic leukemia (CMML) in patients with resistance and intolerance toward hypomethylating agents such as azacitidine and decitabine (Short et al., 2019).

The combretastatins are a family of several cis-stilbenes from Cape bushwillow (*Combretum caffrum,* Combretaceae), a shrub from South Africa. Compounds in the combretastatin class indirectly act on cancer cells by inhibiting tubulin polymerization causing disruption of the tumor endothelial cells lining the tumor vasculature, inducing rapid vascular collapse in solid tumors (Tozer et al., 2002). Combretastatin A1 and combretastatin A4 are the two naturally isolated compounds. Combretastatin A4 phosphate (CA4P) is a phosphate prodrug of combretastatin A4 which has been designated as an orphan drug by the US Food and Drug Administration (FDA) and is approved for the treatment of a range of thyroid and ovarian cancer.

## Conclusions and Future Perspectives

Medicinal plants remain a crucial source in the search and development of new pharmacological leads. One major asset of medicinal plant-based drug discovery is the existence of ethnopharmacological information providing ideal opportunities to limit the huge diversity of possible leads to more promising ones. A novel approach of integrated drug discovery where ethnopharmacological knowledge is supported by broad interdisciplinary forces involving medicinal chemistry, pharmacology, biochemistry, molecular, and cellular biology along with natural product chemistry is necessary to harvest the full potential of phytochemicals. Additionally, the advances in analytical technology and computational methodologies, as well as the development of self-teaching artificial intelligence systems will facilitate the identification of new phytochemical lead entities for pharmacological evaluation.

In the present review, the results observed in different phases of clinical trials along with exciting preclinical results indicate that the ways and means to take phytochemicals “from bench to real-life situations” are on the horizon. In spite of the promise shown by phytochemicals as therapeutic agents in cancer, there are some limitations which need to be resolved. For instance, most of the phytochemicals studied at the preclinical stage lack insight into the molecular interaction with different signaling molecules. To address issues related to molecular targets and pathways, *in silico* strategies like molecular docking need to be employed to understand the interaction of phytochemicals in different signaling pathways that can be further validated by various *in vitro* and *in vivo* models.

In most of the related clinical studies, the presence of methodological flaws including lack of control or placebo group, small sample sizes, and short duration of the trial are observed. Therefore, for many phytochemicals, it is too early to conclude their anticancer actions and hence large-scale and well-controlled clinical trials are needed to validate their efficacies, adverse effects, and safeties before their use for the treatment of cancer. Moreover, extensive standardization in terms of methods for evaluating their bioavailability, efficacy, safety, quality, composition, manufacturing processes, regulatory and approval practices, need to be carried out on the promising phytochemicals to meet the international standard. Paradoxically, vast knowledge and experience in drug development are available in the pharmaceutical industry. Therefore, combining the benefits provided by both traditional and modern medicine has been previously suggested as a promising approach to reveal and to bring new plant-derived substances to market. Synergistic or additional effects of combinations of chemotherapeutic agents and phytochemical compounds in cancer cells with acceptable side effects have been demonstrated (Li et al., 2013; Pezzani et al., 2019). Thus, in recent years, the anticancer and chemopreventive properties of phytochemicals are attracting increasing interest from oncology researchers due to their low intrinsic toxicity in normal cells but prominent effects in cancerous cells (Li et al., 2013).

In this review, an attempt has been made to provide a database of phytochemicals that are used for *in vivo* and clinical studies. This information will be extremely useful to identify a series of additional plant-derived drugs to treat cancer with minimum side effects.

## Author Contributions

OP and PR made substantial contributions to conception and design of the article. AC, PM, and MD contributed to the writing and editing of the manuscript. AC and PM contributed to the designing of the figures and tables. All the authors read and approved the final manuscript for publication.

## Conflict of Interest

Author PR is on the Board of Directors in the company Innovation Biologicals Pvt Ltd, Pune, India.

The remaining authors declare that the research was conducted in the absence of any commercial or financial relationships that could be construed as a potential conflict of interest.
