# Corrigendum: Phytochemicals in Cancer Treatment: From Preclinical Studies to Clinical Practice

**DOI:** 10.3389/fphar.2020.00175

**Published:** 2020-02-28

**Authors:** Amit S. Choudhari, Pallavi C. Mandave, Manasi Deshpande, Prabhakar Ranjekar, Om Prakash

**Affiliations:** ^1^ Combi-Chem Bio-Resource Center, Organic Chemistry Division, CSIR-National Chemical Laboratory, Pune, India; ^2^ Interactive Research School of Health Affairs, Bharati Vidyapeeth Deemed University, Pune, India; ^3^ Department of Dravyaguna Vigan, Ayurved Pharmacology, College of Ayurved, Bharati Vidyapeeth Deemed University, Pune, India; ^4^ Innovation Biologicals Pvt. Ltd., Pune, India; ^5^ Department of Microbiology, Immunology and Parasitology, Louisiana State University Health Sciences Center, New Orleans, LA, United States; ^6^ Stanley S. Scott Cancer Center, Louisiana State University Health Sciences Center, New Orleans, LA, United States

**Keywords:** phytochemicals, anticancer, preclinical, clinical, medicinal plants

In the original article, **Figures 1**–**3** were matched with the wrong legends. The correct figures, with their legends, appear below.

**Figure 1:**
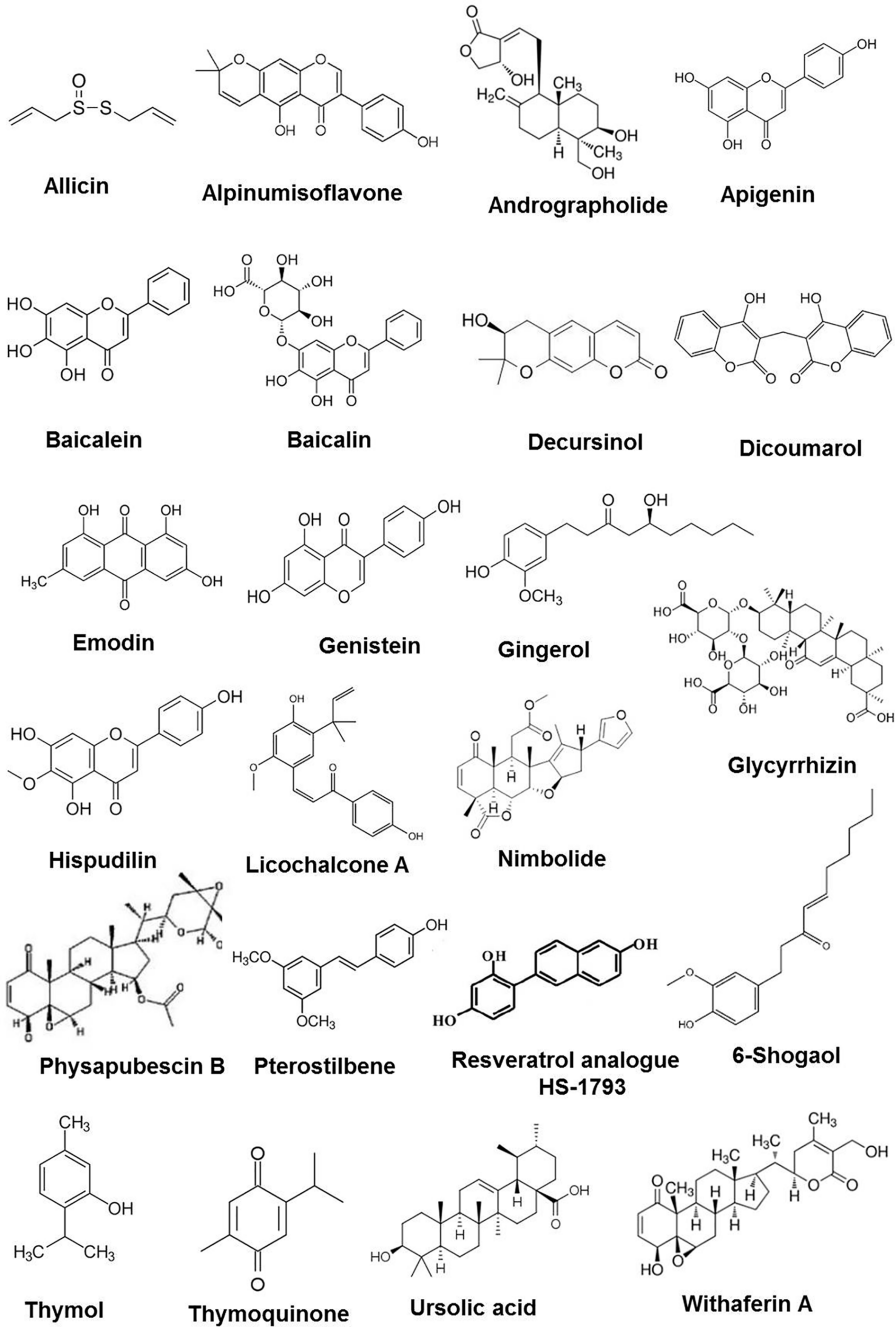
Chemical structures of some anticancer phytochemicals in preclinical trials.

**Figure 2:**
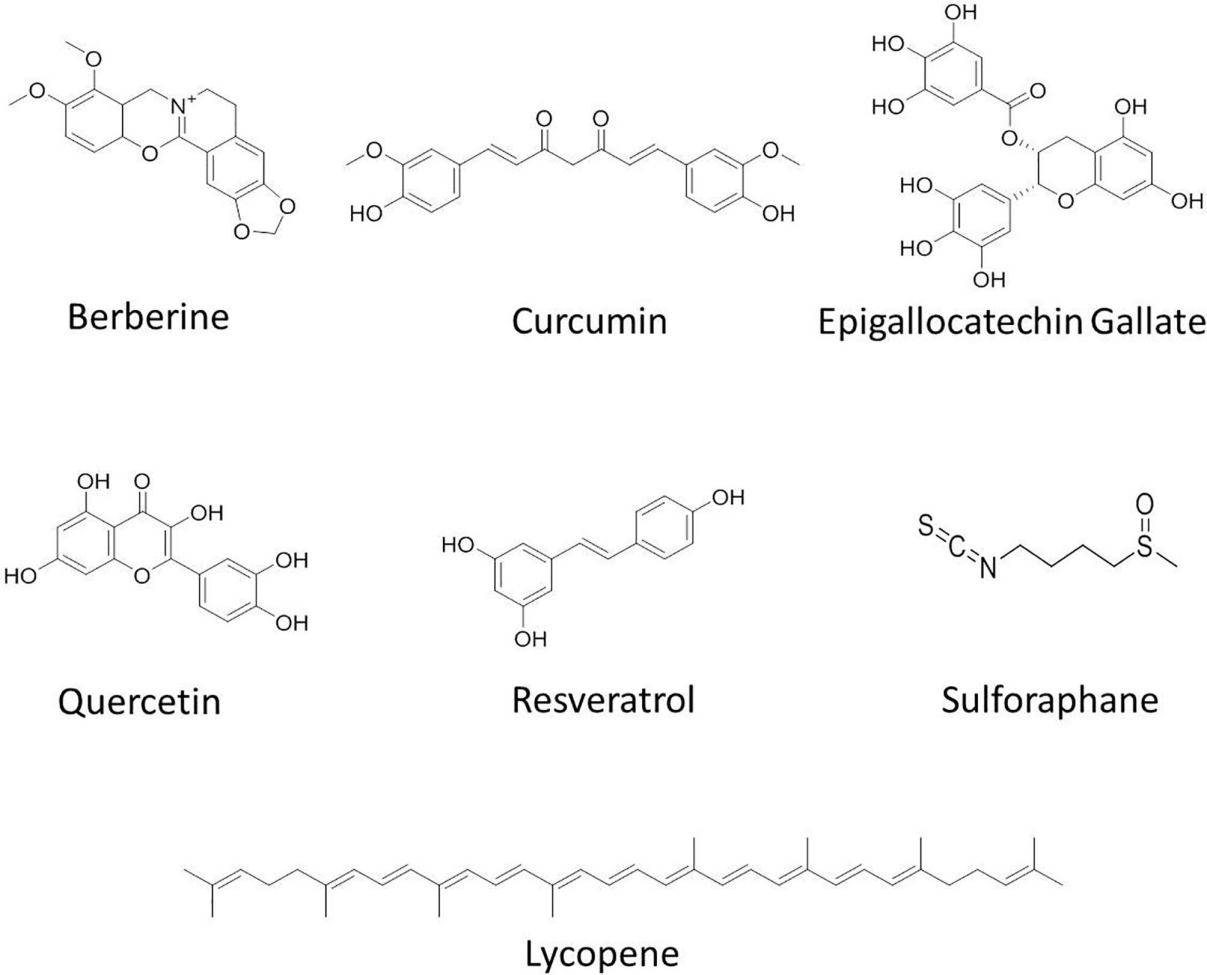
Chemical structures of some anticancer agents in clinical trials.

**Figure 3:**
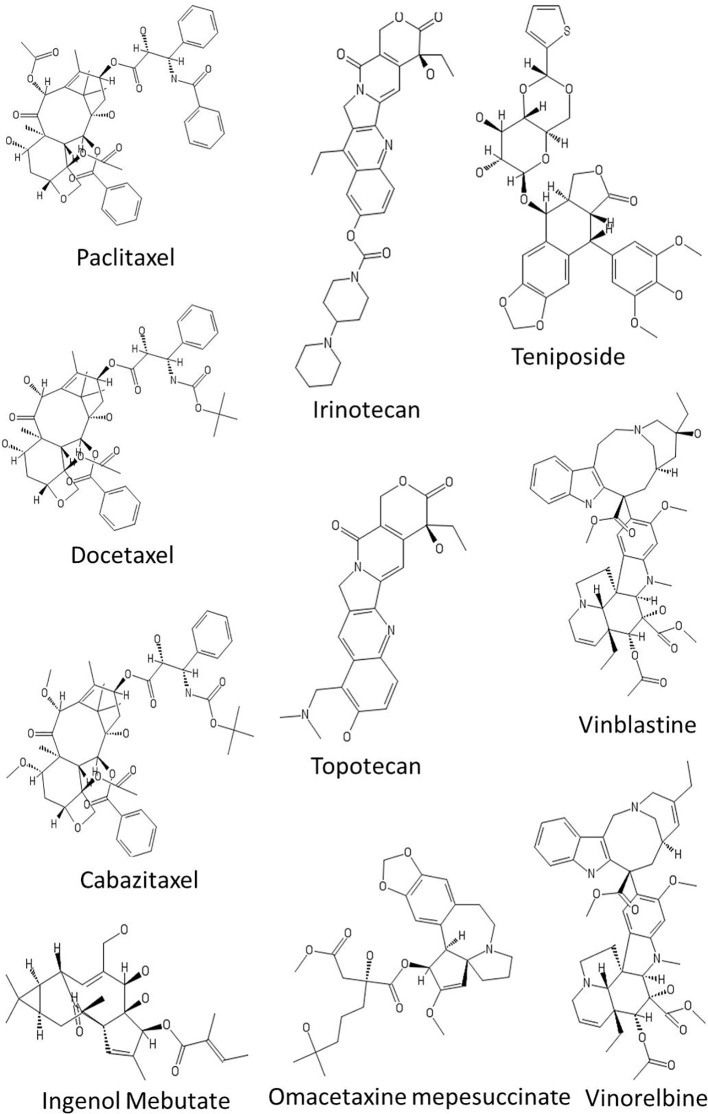
Chemical structures of some anticancer agents in clinical use.

The authors apologize for this error and state that this does not change the scientific conclusions of the article in any way. The original article has been updated.

